# The Present Status of Vaccination1Presidential address delivered at the 68th annual meeting of the Medical Society of the County of Chemung, May 17, 1904.

**Published:** 1904-07

**Authors:** Hamilton D. Wey

**Affiliations:** Elmira, N. Y., President of the Medical Society of the State of New York


### The Present Status of Vaccination.[1]

By HAMILTON D. WEY, M. D., Elmira, N. Y.,
President of the Medical Society of the State of New York.

PLAGUE, pestilence and famine follow in the wake of war; the latter usually immediate and confined to regions contiguous to scenes of conflict, while the two former not infrequently manifest themselves remotely in distant lands and across intervening seas. Following the civil war as troops returned from the front to again engage in the pursuits of private life, smallpox and typhoid fever appeared in communities previously exempt. The same was repeated at the close of the Hispano-American war and upon the return of soldiery from the Philippine Islands. Experience gained during the Civil war conferred upon the practitioner of that period diagnostic acumen in the recognition of variolous disease and skill in its treatment. Knowledge thus obtained was secondarily exercised in civil life with communal benefit and advantage. Certain communities appreciative of preventive medicine exacted a vaccination qualification for participation in school privileges before vaccination was accorded recognition in the statutes of the state, in or about the year ——. Early in the seventies the city of Elmira rigorously required successful vaccination before according admission to the public schools.

Following the declaration of war against Spain and conditions inseparable from and incident to the time, as the massing of large numbers of men collected from all quarters of the country, the establishment of camps, and contact beyond the normal with peoples of tropical countries whose customs, mode of living and traditions differ from our own, variolous disease appeared synchronously in many states. For a third of a century smallpox in the populous portion of the country was a disease of rarity. Sporadic cases carried consternation and apprehension to the communities in which they appeared. During the period intervening between the Civil and Hispano-American wars a generation of physicians had entered upon the practice of their profession, a large majority of whom had never seen a case of smallpox,—to say nothing of never having treated the disease,—who were unfamiliar with it from a clinical standpoint, and whose only knowledge was obtained from cursory reading and a mental picture derived from unsatisfactory and inaccurate prints and illustrations. To these conditions pertaining to the profession may reasonably be ascribed many errors of diagnosis and the injecting into the nomenclature of disease such unscientific and meaningless terms

1. Presidential address delivered at the 68th annual meeting of the Medical Society of the County of Chemung, May 17, 1904.

as Cuban, Spanish, and elephant itch, to say nothing of localisms, and the discrediting of impetigo contagiosa as a morbid entity.

The aftermath of the Spanish war, and the tour of the Joshua Simpkins troupe that terminated ignominiously upon boat quarantine at Geneva, are responsible for the prevalence of smallpox in 1898 and continuing to the present time, extending over an area comprising the central, western and a portion of the southern tier counties of the state, as well as points along the Hudson river and in the Adirondack region. The rôle of war as a disseminator of pestilential disease in civil life has been referred to and mention made of professional shortcomings in diagnosis. In the latter must be included reluctance to diagnosticate smallpox or varioloid.

Uncertain of the correctness of diagnosis in suspected cases it has more than once occurred that physicians were loath to and deferred making a diagnosis repugnant to the patient and his family, on account of the disease itself and the distastefulness of the isolation of quarantine, and unwelcome to the immediate community, the accuracy of which might be questioned subsequently by public opinion or in a court of justice. Thus the true nature of mild cases of eruptive disease has been overlooked until such time as a severe case of smallpox with unmistakable phenomena of the disease declared the true nature of antecedent cases.

There is another reason for the prevalence of smallpox during the past six years. My own opinion is that years ago, so far at least as concerns those of school age, greater protection existed through vaccination than at the present time. Generally speaking, this is particularly true of rural districts. In the days immediately following mandatory vaccination, compliance with the law was more general and unquestioned than now. School boards and directors had regard for the statute and children, as a rule, presented evidence of protection against variolous disease. But time brought a blunting of perception and a gradual growing disregard of preventive medicine, until it came to pass in many sections the law was indifferently enforced and weak-heartedly observed.

My own observation, acquired in an official capacity, is that native born children from adjacent country and a neighboring state are generally unprotected as against smallpox. Then, too, there have arisen Philistines in and out of the profession and certain pseudo medical journals inveighing against vaccination. These have a following who have accepted the intemperate arguments advanced and denunciations uttered as founded upon fact and unanswerable. Their teachings and utterances are in opposition to prophylaxis and treatment of disease by animal derivatives. Agnosticism is by no means confined to matters religious.

It is not necessary in this connection to refer to the objections urged against vaccination because of the alleged production of septicemia, tetanus and the transmission of bovine disease. It is conceded vaccination in all its phases, the initial inoculation and subsequent treatment of the lesion, require as great a degree of attention to details and the principles of asepsis as any surgical undertaking. The use of shields continued after the first twenty-four hours is a crime and cannot be too strongly condemned. Except as above stated they become a vehicle of infection and responsible for sloughing, excessive loss of tissue and septicemia, —conditions that justly discredit and are a reproach to the procedure under discussion. Vaccinal neglect, either by the subject himself or those having him in charge, may lead to untoward results. It was conclusively proved two years ago at Camden, N. J., that the outbreak of tetanus was not due to virus employed but to neglected arms and infection due to contact with dirty hands and finger nails, in a section where tetanus not infrequently appears.

In a measure the country was in a state of preparedness for the smallpox invasion of 1898. Long years of exemption from other than sporadic cases, few and far between, served in a degree to efface the lessons of former years of the beneficence of vaccination. When smallpox finally appeared there was abundance of material susceptible to the disease. The fact that in the recent past smallpox has generally prevailed in a mild form, the number infected being disproportionate to the total number of exposures, does not militate against the statement just made. It is a fact noted in connection with contagious disease, that epidemics occur characterised by a repetition of cases of mild type and feeble power of communication. What causes the materies morbi of infection to repeat itself in malignant form or feeble and benign manner is for others to say. It seems to me it must be evident to those not blindly prejudiced that several states received their just desserts according to the degree they ignored the law and means of prevention.

In this state there is no foundation in law for compulsory vaccination. To subject an individual or child to vaccination against his will and without the consent of parent or guardian constitutes an assault. Health officers and school authorities cannot summarily vaccinate without rendering themselves liable. In the matter of Smith, a Brooklyn case, 146 N. Y., 68, the Court of Appeals decided that no citizen can be compelled to be vaccinated against his will. To do so by force would be an assault. Nor can a person be quarantined simply for refusing to be vaccinated. In the case above referred to, one Smith lived in an infected district and was engaged in the local truck and express business. He refused to be vaccinated and was quarantined by the health officials. This was held illegal, the court deciding the only grounds for quarantine were an actual infection with the disease or an actual exposure to it. The mere possibility of exposure does not justify a quarantine.

Section 200, Article XII., Chapter 661 of the Laws of 1893, commonly spoken of as the public health law, provides: "No child or person not vaccinated shall be admitted or received into any of the public schools of the state, and the trustees or other officers having the charge, management or control of such school shall cause this provision of law to be enforced." The law provides for the adoption of a resolution excluding unvaccinated children and persons from school until vaccinated, and the posting of the resolution in at least two public and conspicuous places within the school district. Provision is also made for vaccination at public expense of any child or person of suitable age desirous of attending school, whose parents or guardian are unable to procure vaccination for them. There is no ambiguity of expression as to the intent of the law—namely, that no unvaccinated child or person shall be received or admitted into any public school of the state. The constitutionality of the law has been attacked, but the courts have upheld the law and declared in effect that while the constitution of the state imposes upon the legislature "the maintenance and support of a system of free common schools, wherein all the children of the state may be educated," such common school system is not to be regarded as a constitutional right, but simply a privilege.

The reference in the constitution to the public school system is for the purpose of safeguarding and continuing an existing privilege. The public school system being then a privilege, it is competent for the legislature to impose a qualification for participation in such privileges as shall operate equally and without distinction upon all who are, or may desire to become, pupils in the public schools. Such qualification is vaccination applicable to and affording protection to all pupils. The law states that children under a certain age shall attend school; otherwise they render themselves amenable to the provisions of the truant law. The question suggests itself, what disposition shall be made of children becoming truants through the act of parents or guardians in refusing vaccination, either by reason of prejudice or upon the ground of conscientious scruples? To my knowledge this matter has not been passed upon by the courts.

Edmund C. Viemeister, of the Borough of Brooklyn, sought to compel the principal of Public School No. 12 to admit his child to the school, admission having been denied on account of the child not having been vaccinated. It was urged in behalf of Viemeister that the statute enforcing the vaccination of public school children was void, being contrary to the provision of the constitution. Under date of November 20, 1903, the Appellate Division of the Supreme Court for the second department, sitting in Brooklyn, upheld the constitutionality of the statute. The prevailing opinion by Mr. Justice Woodward is substantially as follows: Section 1 of Article IX. of the Constitution of the State of New York, which says: "The legislature shall provide for the maintenance and support of a system of free schools wherein all the children of the state shall be educated" did not operate to make education a constitutional right rather than a privilege, and it was intended merely to secure a continuance and an extension of an existing privilege. The legislature has the right to impose any reasonable regulations upon the exercise of this privilege, provided that such regulation operates equally upon all persons in the same class and under the same conditions, and Section 22 of the Public Health Law (Laws of 1893, Chapter 661,) which prohibits children from attending the public schools without first having been vaccinated, is a legitimate exercise of this power of regulation, and is constitutional. Such section does not violate the constitutional provision that no citizen shall be deprived of any of his rights and privileges "unless by the law of the land or the judgment of his peers." An appeal was taken from this order of the Supreme Court, made at the Kings County Special Term and entered in the office of the clerk of the County of Kings on the 14th day of July, 1902, denying the petitioner's motion for a peremptory writ of mandamus. Justice Herschberg also wrote an opinion sustaining the statute, declaring it constituted a legitimate exercise of the police power of the state.

Instances were not lacking this past winter of rural school boards deliberately refusing to enforce the law relating to vaccination. Nor does it appear that peremptory closing of public schools in certain sections quickened obtunded faculties and blunted sense of duty. "No child or person not vaccinated shall be admitted or received into any of the public schools of the state." The law notes no exception and excuses none from its specific provision. There has, however, come into practice the exercise of arbitrary discretion on the part of school authorites in consideration of the alleged ill health of a child, inability to withstand the shock of a minor surgical procedure, and preëxisting cutaneous lesion that might possibly be aggravated by vaccination, and claimed immunity to vaccine virus demonstrated by one or more previous futile attempts. Usually, there is little or no justification —more frequently none than any,—and that but slight, for such attempts and subterfuges to evade the law. The assumption of such discretion is illegal, unwarranted and unjustified. From a medical point of view a child has either been vaccinated or it has not. If the former, it is eligible to the public schools in conformity with the public health law. In the latter case it follows that, through intent or by reason of imperfectly demonstrated non-susceptibility, the child has never experienced the lesion and phenomena following successful inoculation with the virus of cowpox or so called humanised vaccine. One, two, three or more attempts unsuccessfully performed do not constitute a vaccination or indicate immunity to either or both vaccinia and smallpox. Unsuccessful attempts may constitute vaccination from the pedagogic point of view, especially when a large enrollment of scholars is desired; but from a medical and clinical standpoint, never.

I am compelled, however, in justice to school authorities to say that they are justified by legal opinion in the course occasionally taken. The view expressed by the Attorney General upon this point is simply an opinion, not a decision of the court; and not being a decision by the court cannot be regarded as a final and conclusive interpretation of the statute. Under date of February 18, 1904, I wrote the State Commissioner of Health as follows: "Can you refer me to any decision, or officially inform me what constitutes a vaccination? Does vaccination imply: (1) a successful effort from a clinical point of view? (2) do a number of attempts (3 or 4), upon the same individual, all unsuccessful, constitute a legal vaccination and permit of the attendance of such a one upon the public schools?" The questions raised were submitted to the Attorney General who replied February 24, as follows: "The object of vaccination is the prevention of smallpox. The method employed is inoculation with the virus of cowpox. It is the common experience of mankind that cases are of frequent occurrence in which the act of inoculation is not followed by swelling, soreness and inflammation, which are the natural and generally expected results of vaccination. In such cases there is a strong probability that the particular subject is immune, either as the result of a former vaccination or for some other cause perhaps not clearly understood. In my judgment the vaccination called for by the statute is accomplished when the patient has been inoculated with the virus of the cowpox."

Until such time as the courts shall have passed upon the question under immediate consideration I presume school authorities, at least those mindful of the provisions of the public health law, will govern themselves according to law. But it is incumbent upon them to proceed slowly and cautiously in accordance with the advice given by the state's legal adviser. The opinion given may pass as legal advice, but I feel I am warranted in saying there is not a physician of standing and experience in Chemung County but will dissent from the utterances and conclusions of the Attorney General. They are based upon misunderstanding and false premises, are erroneous and constitute a menace to public health. Such an opinion from other than the Attorney General of the state of New York would command a smile or a look of compassion from any one of you. Those of us who are engaged in public health service, interested in the administration of health laws, and with the best interests of the public schools at heart, are disappointed that such an opinion should have been received from such a source and made public. It is a blow to preventive medicine and paves the way for artifice (nitric acid traumatism), fraud and false representation. Physicians are not wholly blameless in conveying the impression a child is immune to vaccination. Two or three ineffectual attempts, possibly faulty technic, unreliable virus, a weak yielding to the importunities of parents, and a child is declared insusceptible to vaccination—and a certificate accordingly given. One, two or three attempts do not demonstrate vaccinal immunity. Error may lie with the operator; the virus employed may be inert. It is safer to err in the opposite extreme than exhibit undue haste in certifying for the purpose of obtaining admission to the public school a child cannot be successfully vaccinated. Recently I successfully vaccinated a child upon whom the operation had previously been twice performed and admitted to school upon such representation. In another instance two years and frequent attempts, six or seven, were required before the child conformed to school requirements. This phase of my topic has been rendered perplexing to the physician by reason of the unsatisfactory, worthless and inert virus upon the market for some time past. For two or more years, so-called vaccine virus has proved a disappointing agent in my hands. I speak impartially and without prejudice for I have used all forms of the commercial article sold in this city. The physician who is able to attain 98 per cent. success in primary cases with commercial virus, such as we have had for two years is a genius, and it would afford me pleasure to look upon his countenance. Such results are exploited and referred to as demonstrating the superiority of this virus or that. With virus thus advertised I have never been able to more than reach the outer limit of the shadow of such success. Within a short time the agent of a supply house confessed to me the vaccine product of his firm had proved unstable and deficient in results.

He could assign no cause, but derived the comfort due him from the consciousness his competitors fared no better. Whether cow-pox has largely lost its former specific property through continued cultivation and transmission, and further modified by methods of preparation for the market, are laboratory propositions. I cannot regard it as altogether a happy and fortuitous day when bovine superceded humanised virus. There is much to commend the former—you are as well aware of this as I. But I am of the opinion, and believe the older practitioners will sustain me in it, that accidents of sepsis, from conditions inherent in the virus and communicated disease in former days were of no greater frequency when humanised virus was generally employed than in these latter times when bovine virus has attained the position of a fetich. The times change and we change with them; but it does not follow that viewed in the light of today the past was altogether an error.

We have convened today in observance of the sixty-eighth annual meeting of the Medical Society of the County of Chemung. We have not been called upon the past year to record the passing of any members, nor have matters medical of unusual importance relating to administrative questions presented themselves for consideration. The year has been uneventful and harmonious. I desire to express my obligations to those who through their coöperation and presentation of papers of value have conferred interest upon our meetings. Whatever the measure of success attained, has been due to their efforts, and I here record my appreciation. In every instance my requests have met with hearty and cheerful compliance.

I hoped to be able to announce at this meeting the union of the two state medical organisations and a united profession. But matters that seemed of sufficient importance to the joint committee charged with preliminary details have delayed final action. It is confidently believed that before we meet again consolidation of the societies will be an accomplished fact, past differences reconciled, and the profession united upon a sound and substantial basis.

359 MAIN STREET.

---

RHEUMATIC DYSMENORRHEA.

| | | |
|---|---|---|
| ℞ | Tr. Cimicifugæ | ℥ii. |
| | Tr. Stramonii | ℥ss. |
| | Tongaline | ℥iv. |

M. Sig.—A teaspoonful in water at meal times.

